# ASO Author Reflections: Emerging Risk Factors in Colon Cancer—End of the Line for Clinomics?

**DOI:** 10.1245/s10434-019-08149-2

**Published:** 2019-12-28

**Authors:** Erik Osterman, Bengt Glimelius

**Affiliations:** 1 Department of Immunology, Genetics and Pathology, Uppsala University, Uppsala, Sweden; 2 Department of Surgery, Gävle Hospital, Gävle, Sweden; 3 Department of Oncology, Uppsala University Hospital, Uppsala, Sweden

## Past

The risk of recurrence after colon cancer surgery today is less than seen in the past, likely ascribed to higher quality in staging, surgery, and pathology.^1^ Further stratification of patients is needed to guide treatment decisions, especially in the light of the IDEA collaboration findings.^2^ Many clinicopathological risk factors of recurrence have been suggested over the years but not always tested with the routinely used risk factors suggested in guidelines.^3^ We sought to test routinely collected, emerging, risk factors not incorporated in guidelines in a well-characterized cohort against the baseline of clinicopathological data and risk factors suggested in NCCN guidelines.

## Present

We found that all emerging risk factors correlated with worse clinical features and with recurrences. However, after adjusting for T- and N-stage, and NCCN risk factors it appears obvious that several risk factors are symptoms of advanced disease and not independent risk factors. In unadjusted analyses pT4a was worse than pT4b, replicating the results from our national Swedish registry study. Carcinoembryonic antigen (CEA) was previously recommended as a risk factor before surgery. We found that if CEA was elevated after surgery, but before initiation of adjuvant chemotherapy, as many as 48% recurred compared with 16% if not.^4^

## Future

Testing CEA after surgery, but before chemotherapy, should be investigated further, because it could help to better stratify patients. The finding of worse outcomes for pT4a than pT4b patients suggests that the terminology for pT4 subclassification may need to be reversed, again, to preserve the logic of the system, if the finding holds.

Future attempts to improve risk stratification of patients with colon cancer should consider clinomics, potentially with additional evaluation of the histopathological pattern, and adjust for them, but look further into molecular and genomic factors. Predicting treatment response and evaluating residual disease is currently a hot topic, with ctDNA taking a strong position.^5^ Risk stratification with clinomics and biomarkers for treatment decisions, determination of residual disease for treatment length and intensity of follow-up could be the future.
